# Urban–rural linkages: effective solutions for achieving sustainable development in Ghana from an SDG interlinkage perspective

**DOI:** 10.1007/s11625-021-00929-8

**Published:** 2021-03-09

**Authors:** Gideon Baffoe, Xin Zhou, Mustafa Moinuddin, Albert Novas Somanje, Akihisa Kuriyama, Geetha Mohan, Osamu Saito, Kazuhiko Takeuchi

**Affiliations:** 1 GCRF Centre for Sustainable, Healthy and Learning Cities and Neighbourhoods (SHLC), School of Social and Political Sciences, University of Glasgow, Rm 710 Adam Smith Building, 40 Bute Gardens, Glasgow, G12 8RS UK; 2 Institute for Global Environmental Strategies (IGES), 2108-11 Kamiyamaguchi, Hayama, Kanagawa 240-0115 Japan; 3 United Nations University Institute for the Advanced Study of Sustainability (UNU-IAS), 5 Chome-53-70 Jingumae, Shibuya City, Tokyo, 150-8925 Japan; 4 The University of Tokyo Institute for Future Initiatives (IFI), 7-3-1 Hongo, Bunkyo, Tokyo, 113-0033 Japan

**Keywords:** Urban–rural linkages, Sustainable development goals (SDGs), SDG interlinkage analysis, Integrated policymaking, Ghana

## Abstract

Urbanization and concomitant challenges pose a great threat to sustainable development. Urban and rural development interacts through the flows of people, materials, energy, goods, capital, and information. Without building sound urban–rural linkages, achieving development in one area could compromise it in another area. Achieving sustainable development needs customized policy prioritization and implementation in both urban and rural areas. Much literature exists in the research field of urban–rural linkages, but little has been done via a comprehensive analysis from an interlinkage perspective in the context of the Sustainable Development Goals (SDGs). Sustainable Development Goal 11 on sustainable cities and several targets embedded under other Goals provides a good framework for analyzing the urban–rural linkages. This paper contributes to this novel research perspective using Ghana as a case. The study applied an integrated approach by combining the results from a solution-scanning exercise with an SDG interlinkage analysis to identify the challenges and priority solutions and assess the synergies and trade-offs of the identified solutions. It extends the conventional solution-scanning approach by further assessing the synergies and trade-offs of the solutions from an SDG interlinkage perspective. It also enables a more practical SDG interlinkage analysis through the contributions from the multi-stakeholder consultations conducted in Ghana. The analyses show that prioritizing gender inclusion (Goal 5) will positively affect many social and well-being outcomes, including poverty elimination (Goal 1), hunger reduction (Goal 2), health improvement (Goal 3) and access to quality education (Goal 4) and basic services, such as water (Goal 6). However, gender inclusion could have potential trade-offs in the agricultural sector (Goal 2) in the case that women who dominate agricultural value chains could move to work in other sectors. Lack of proper infrastructure (Goal 9), such as transport, will hinder wide gender inclusion. An integrated approach that considers both the synergies and trade-offs of relevant solutions is critical for effective policymaking, specifically in developing countries.

## Introduction

Urban–rural linkages are defined as the cultural, economic, environmental, social, and political connections between communities on the urban and rural divide (Tacoli 2002). Braun (2007) and Tacoli (1998, 2003) defined urban–rural linkages as the spatial flows of individuals, merchandize, money, social values, and sectorial flows, such as agriculture and non-agricultural employment between urban and rural areas. It is defined as the flows between labor markets and migration, services, resources, and information, and connecting institutional functions such as partnerships at various levels (local, national, and international) (Smith and Courtney 2009).

The concept of urban–rural linkages is significant to poverty alleviation work and the efforts for achieving wider equality made by many development organizations, such as the Organization for Economic Cooperation and Development (Tacoli 2004), the World Bank (Christiaensen and Todo 2014), the Department for International Development (Farrington 2002), New Urban Agenda (Habitat III) (United Nations 2017a, b), and the International Monetary Fund (Adam et al. 2016). Rural and urban areas are linked by agricultural value chains and food systems and by ecosystem services, labor, natural resources, energy, transport, and intermediate towns (Hussein and Suttie 2016). Meeting the globally growing demand for food, especially in urban areas, and the conservation of ecosystems and their services are contingent on a thriving sustainable agriculture and the development of rural areas (Hussein and Suttie 2016). While significantly influencing the creation of wealth, welfare, and employment, urban–rural linkages are also critical for improving regional governance and the competitiveness of related sectors and regions (Lucatelli and De Pietro 2013; Calì and Menon 2012).

In Ghana, the urban–rural dichotomy has been a major challenge for governmental policies. Efforts to raise living standards and enhance economic growth have resulted in urban-biased policies inadvertently and unequal spatial development patterns. Currently, extreme disparities in demographic and settlement patterns, social infrastructure, and the levels of economic development exist. This skewed development trajectory resulted in substantial differences between urban and rural settings in, for instance, the distribution and quality of education facilities and human resources (Opoku-Asare and Siaw 2015). Rural areas in Ghana, unlike their urban counterparts, are characterized by agriculture and informal economies with limited access to economic, physical, and human assets (Boakye-Yiadom 2008; Baffoe et al. 2014; Baffoe and Matsuda 2015, 2017b, c), and a declining environmental quality because of overdependence on natural resources (Baffoe and Matsuda 2017a, 2018). This situation has made rural areas in Ghana unattractive, especially among the youth, with a net effect of out-migration and associated negative impacts on agricultural labor and productivity. Many people, particularly the youth, continue to move to the major cities (e.g. Accra, Kumasi and Takoradi) in search of better livelihood options (Nyame et al. 2009; Abu et al. 2014; Awumbila et al. 2014). Urbanization level, for instance, increased by 7% from 43.8% in 2000 to 50.9% in 2010 (Ghana Statistical Service 2010). Although internal migration is largely premised on economic gains, studies have shown that poverty among migrants in Ghana have worsened in most cases, as many are challenged to secure a job (Awumbila et al. 2014; Imai et al. 2017; de Brauw et al. 2014; NDPC 2017). Bridging the urban–rural divide across the country, especially in the education sector, will be critical in unleashing both development and growth potentials (Opoku-Asare and Siaw 2015). Context-specific attempts to narrow the gap include the development and implementation of decentralization policies and systems. Since the passage of the Local Government Act in 1993, successive governments have worked assiduously to devolve powers and functions to the regional and district levels (Resnick 2018), with the underlying motive to spur rural growth through effective urban–rural linkages (Owusu 2004, 2005). Importantly, Ghana’s decentralization policies aim to promote citizenry participation in decision-making, agriculture development, improvement in income and poverty reduction (Owusu and Lund 2004; Owusu 2005). The current one-district-one-factory initiative (https://1d1f.gov.gh/) is another major strategy aiming to stimulate economic development and rural development across the country. Also important is the National Urban Policy (NUP) Framework and Action Plan, which seeks to promote effective urban–rural linkages by establishing rural service centers and promoting agriculture activities and the development of agro-based industries (Ministry of Local Government and Rural Development 2012).

Studies on urban–rural linkages have focused on multiple issues, such as migration; remittances; social services, including health and education; nutrition; climate change; and food security (Fotso 2007; Mizéhoun-Adissoda et al. 2016; Trotter 2016; Bishwajit and Kpoghomou 2017; Allegretti 2018). Meanwhile, existing literature has paid little attention to the challenges and policy solutions of urban–rural linkages. The work conducted by Somanje et al. (2020), using a stepwise solution-scanning technique, identified urban–rural challenges and associated solutions through a comprehensive literature review. They further screened and ranked the solutions based on the stakeholder consultations conducted in Ghana. This study, building on the work by Somanje et al. (2020), conducted a quantified interlinkage analysis of Sustainable Development Goals (SDGs) to identify the synergies and trade-offs of the top solutions with other SDGs relevant to urban–rural linkages.

Because of the complex and profound interactions between cities and rural areas, achieving development in one area could compromise it in another area. Achieving sustainable development requires customized policy prioritization and implementation in both urban and rural areas by building effective linkages between the two. The SDGs, covering a standalone Goal 11 on sustainable cities and several targets embedded under other Goals, provides a relevant framework for analyzing urban–rural linkages. However, SDGs and associated targets represent a broad set of diverse elements that are also intrinsically interconnected, and they can be mutually reinforcing or conflicting. Unlike the conventional silo-based approach for development, SDGs should take an integrated approach (United Nations 2015a). Therefore, policymakers face a range of cross-area integration and collaboration processes with which they are unfamiliar. Understanding how the SDGs interact is, therefore, crucial for integrated and effective decision-making (Nilsson et al. 2016, 2018; ICSU 2017; Pradhan et al. 2017; Weitz et al. 2017). These interactions include both synergies and trade-offs. For instance, improving electricity access (Goal 7) will positively affect students’ studying time (Goal 4), but if such electricity access is achieved by the increased use of fossil fuels, it could adversely affect a country’s climate mitigation efforts (Goal 13). However, if renewables are used to enhance energy access, it would help in reducing dependency on solid fuels and kerosene for cooking and lighting (Goal 7), also helping in curtailing indoor air pollution and associated respiratory diseases (Goal 3) (Collste et al. 2017). Therefore, achieving the goals demands an integrated assessment to maximize the synergies and minimize the trade-offs to support a pragmatic policy formulation (van Soest et al. 2019). Even though the 2030 Agenda calls for an integrated approach, the Agenda and related policy processes do not elaborate on how goals/targets are interconnected. Given this, the International Council for Science (ICSU) has called for approaches and strategies to support interlinkages analysis to aid in policy design and implementation strategies (ICSU 2017).

Although it is critical to understand the synergies and trade-offs of the SDGs for policymaking, knowledge on the interactions among various goals and targets is limited, presenting a major research gap (Weitz et al. 2017). A robust analytical tool for analyzing SDG interactions is also lacking (Griggs et al. 2014; Nilsson et al. 2018). A few existing studies have used different approaches to explore these interactions, including Integrated Assessment Models (IAMs) (Dickson et al. 2010; Nerini et al. 2017; van Soest et al. 2019), influence matrix (Scharlemann et al. 2020), and typology characterization (Nilsson et al. 2018; Jiménez-Aceituno et al. 2020). The system-based approaches, initially applied by Le Blanc (2015) in the context of SDGs, could help in understanding the interactions among the SDGs using social network analysis techniques. The SDG Interlinkages Analysis & Visualisation Tool (briefly called as the SDG Interlinkages Tool), developed by the Institute for Global Environmental Strategies (IGES), is among the currently available system-based tools. The tool provides a methodology to identify the linkages between relevant SDG targets based on causation and then quantify the linkages using country-specific indicator-level data (Zhou and Moinuddin 2017). The tool currently covers 27 countries in Asia and Africa, including Ghana. Given the tool’s suitability for this study, it was used for an interlinkage analysis to assess the synergies and trade-offs of the top solutions identified by Somanje et al. (2020) to support recommendations on effective solutions for building effective urban–rural linkages.

## Methods

**Fig. 1:**
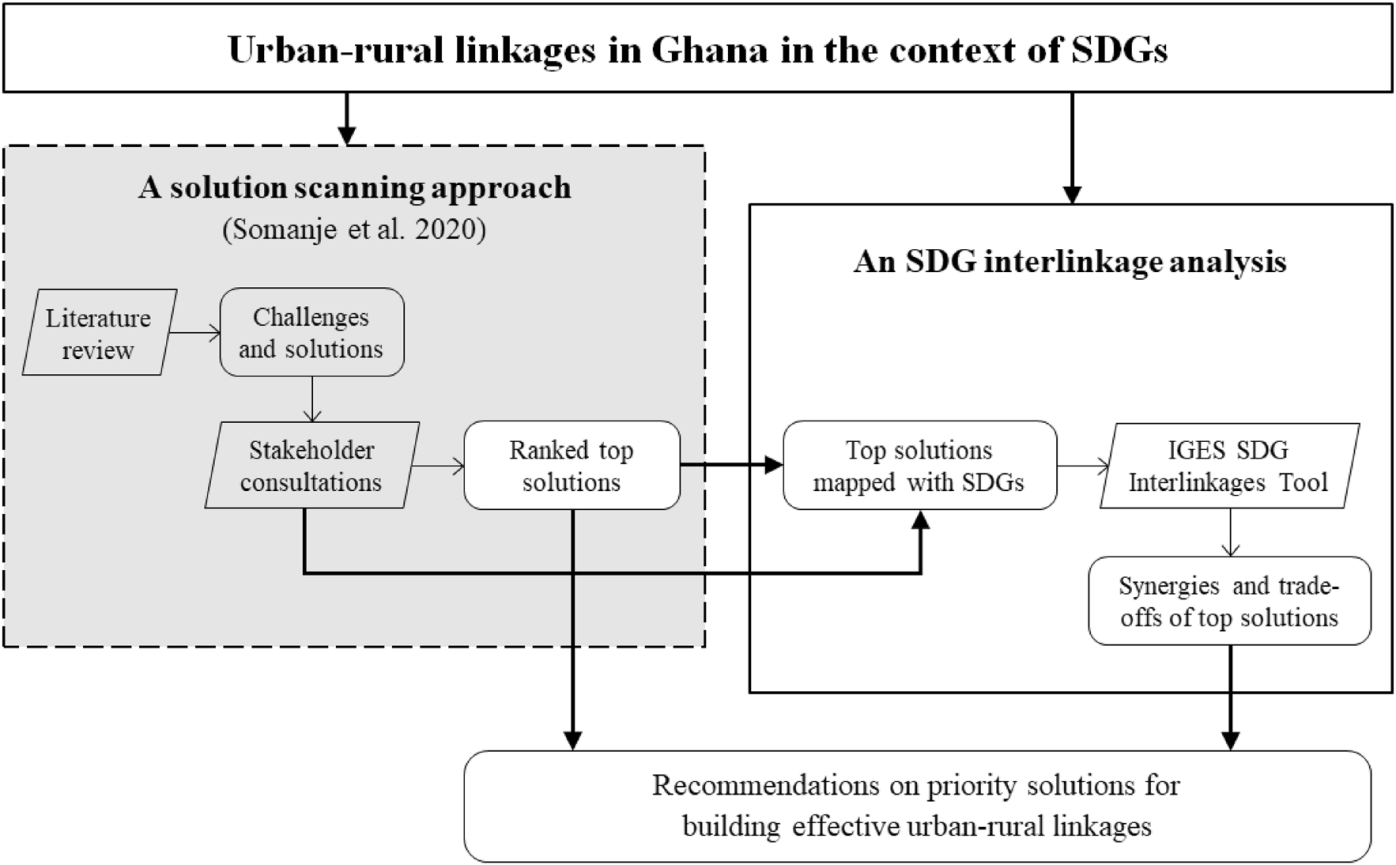
An integrated approach for the study on urban–rural linkages in Ghana from a perspective of SDG interlinkage

For the study of the urban–rural linkages in Ghana in the context of SDGs, an integrated approach was applied by incorporating the results from the solution-scanning exercise conducted by Somanje et al. (2020) into an SDG interlinkage analysis (Fig. 1). Somanje et al. (2020) used a solution-scanning approach to identify the challenges and solutions for building effective urban–rural linkages. A solution-scanning exercise was conducted by performing a comprehensive literature review to identify the challenges and solutions. Through a series of multi-stakeholder consultations, the identified challenges and solutions were further screened and ranked to find top-priority solutions (see the dotted frame in Fig. 1).

Solution scanning is a technique of finding a list of potential policy actions, interventions, solutions or methods that respond to a broad challenge (Sutherland et al. 2014; Hernández-Morcillo et al. 2018). Identifying the list helps policymakers to formulate pragmatic interventions (Sutherland et al. 2014; Dicks et al. 2017; Hernández-Morcillo et al. 2018). According to Sutherland et al. (2014), a strategic and comprehensive review and identification of potential solutions are critical, as it provides a wide range of possibilities before one will narrow down to the selected options. This makes it easier to keep track of unselected options in the subsequent steps. The approach has been employed in many areas such as environmental scanning (Guion 2010), identification of scientific research agenda (Sugiyama et al. 2017), conservation prioritization (Pullin et al. 2013), ecosystem services (Sutherland et al. 2014), food networks (Plieninger et al. 2018) and climate change adaptation and mitigation (Hernández-Morcillo et al. 2018). More recently, Somanje et al. (2020) used solution scanning to explore the challenges and solutions to address urban–rural linkages in Ghana.

In this study, based on the top four solutions identified by Somanje et al. (2020), an SDG interlinkage analysis was further conducted to understand the synergies and trade-offs that the top solutions might generate among themselves and with other SDG targets. In doing so, the identified top four solutions were mapped with relevant SDG targets based on the results from the multi-stakeholder consultations conducted in Ghana (Somanje et al. 2020). Next, using the SDG Interlinkages Tool, the interlinkages of the mapped SDG targets were analyzed for Ghana. Consequently, the synergies and trade-offs of the mapped SDG targets of top solutions were identified. By linking the solution scanning exercise based on a literature review and stakeholder consultations and the SDG interlinkage analysis based on a quantitative analysis and visualization, the potential solutions can be identified, screened and ranked, and their synergies and trade-offs can be assessed. The identified top solutions together with the information on their synergies and trade-offs can be used to inform effective urban–rural planning and priority setting.

By integrating the solution scanning approach and the SDG interlinkage analysis, the novelty of this study to the area of solution scanning is to extend with a further assessment of the synergies and trade-offs of the screened solutions through an SDG interlinkage perspective. The value-added of this study to the area of SDG interlinkage analysis is to enable a problem-and-solution-driven exercise through which stakeholder consultations provide invaluable inputs to screening the top solutions and mapping with SDGs.

### Identification of top solutions for building effective urban–rural linkages in Ghana and mapping with SDGs

Somanje et al. (2020) identified four top-priority solutions for building effective urban–rural linkages in Ghana based on a literature review and multi-stakeholder consultations. They further discussed the implications for achieving sustainable urban and rural development in Ghana. The four top solutions include: (1) gender inclusiveness, which is equal participation of male and female in decision-making in social, economic, and political development; (2) investment in basic and economic physical infrastructures, such as integrated transport systems (water, rail, and roads), and compact city concept for high-rise buildings to maximize land use efficiency; (3) modernization of agricultural systems to enhance sustainable agricultural practices and food production systems through technological innovation (such as the integration of ICT in extension services, precision agriculture, formalization of urban agriculture, food safety, and nutrition) and institutional changes (such as reorientation of curriculum, extension, and research and development to meet the current challenges of the food system); and (4) innovative financial inclusion to enable urban and rural poor to have access to affordable financial services, banking/credit facilities, and quick services for remittances such as mobile money accounts. The four top solutions were further mapped with relevant SDG targets based on the results from the multi-stakeholder consultations conducted in Ghana (Somanje et al. 2020). Table 1 lists four top solutions and their mapped SDG targets. The list of 17 SDGs is provided in Appendix 1.

**Table 1:** Top solutions for building effective urban–rural linkages in Ghana and mapping with SDGs

Top solutions	SDG mapping	Indicators	Data sources^a^
Gender inclusiveness	3.7 Universal access to reproductive healthcare services	3.7.1 Proportion of women married or in a union of reproductive age (aged 15–49 years) who have their need for family planning satisfied with modern methods (% of women aged 15–49 years)	Ghana 2003 Demographic and Health Survey
	4.5 Eliminate gender disparities in all levels of education	4.5.1 Gender parity index for participation rate in organized learning (one year before the official primary entry age), (ratio)	UIS
	5.1 End gender discrimination	Unavailable	
	5.5 Enhance women’s participation in decision-making and public life	5.5.1 Proportion of seats held by women in national parliaments (% of total number of seats)	Inter-Parliamentary Union, the database on Women in National Parliament, accessed February 1 of given year for annual data
	5.a Women equal rights to economic resources	Unavailable	
	5.b Using technologies for women empowerment	Unavailable	
	10.2 Promote social, economic, and political inclusion of all	Unavailable	
	10.4 Policy for greater equality	Unavailable	
Investment in basic services and economic infrastructure	2.1 End hunger	2.1.1 Prevalence of undernourishment (%)	FAO, Statistics Division
	3.8 Universal health coverage	Unavailable	
	4.1 All for free primary and secondary education	4.1.1 Minimum proficiency in mathematics by education level and sex (%)	TIMSS 2003
	6.1 Universal access to safe drinking water	6.1.1 Proportion of population using safely managed drinking water services, by urban/rural (%)	WHO/UNICEF Joint Monitoring Programme for Water Supply, Sanitation and Hygiene (2017)
	6.2 Universal access to sanitation and hygiene	6.2.1 Proportion of population practicing open defecation, by urban/rural (%)	WHO/UNICEF Joint Monitoring Programme for Water Supply, Sanitation and Hygiene (2017)
	7.1 Universal access to energy	7.1.1 Proportion of population with access to electricity, by urban/rural (%)	Source: Global Tracking Framework 2018
	9.c Universal and affordable access to ICT	9.c.1 Proportion of population covered by a mobile network, by technology (%)	ITU estimate
	10.c Reduce the transaction costs of migrant remittances	10.c.1 Remittance costs as a proportion of the amount remitted (%)	World Bank. World Development Indicators database
	11.1 Universal access to urban housing and basic services	11.1.1 Proportion of urban population living in slums (%)	United Nations Human Settlements Programme (UN-HABITAT)
Promotion of sustainable agriculture systems	2.3 Double agriculture productivity	2.3.1 Agriculture, forestry, and fishing, value added per worker (constant 2010 US$)	World Bank. World Development Indicators database
	2.a Investment in agriculture extension programs	2.a.1 Agriculture orientation index for government expenditures	Unavailable
Innovative financial inclusion systems	8.10 Strengthen financial institutions	8.10.1 Number of commercial bank branches per 100,000 adults	Financial Access Survey (FAS), IMF's Statistics Department, accessible at http://imf.data.org/FAS
	9.3 Increase access to financial services	9.3.2 Proportion of small-scale industries with a loan or line of credit (%)	World Bank Enterprise Surveys 2018
	10.7 Improve equality of migrants	Unavailable	
	10.c Reduce the transaction costs of migrant remittances	10.c.1 Remittance costs as a proportion of the amount remitted (%)	World Bank. World Development Indicators database

^a^For the details of the data sources, please refer to the SDG Interlinkages Tool V3.0 (Zhou et al. 2019)

In Table 1, the indicators for the mapped SDG targets are based on the SDG Interlinkages Tool, which uses the Global SDG Indicators (UNSD 2018) and other global indicators (e.g., the World Bank’s World Development Indicators). This was necessary due to the considerations of compatibility and comparability across countries. It should be noted that in some cases using national and local indicators might be more relevant to reflect specific context at different scales. For example, besides Indicator 5.5.1, “proportion of seats held by women in national parliaments”, the indicator of “the proportion of women as Mayors and heads of rural districts” which is available in Ghana can be more relevant for the analysis of the urban–rural analysis. In addition, given that mobile money and integrated digital platforms for financial transactions and savings, as well as non-banking institutions such micro-finance and savings, are available in many developing countries including Ghana, using Indicator 8.10.1, “number of commercial bank branches per 100,000 adults”, may greatly underestimate the proportion of the population which is financially inclusive. The existing gaps in the selection of indicators with trackable data for measuring relevant SDG targets at different scales constrained the utility of the SDG interlinkage analysis to address context-based synergies and trade-offs.

### SDG interlinkage analysis

The SDGs includes a standalone Goal 11 on sustainable cities with 10 targets covering the issues of safe and resilient human settlements, sustainable transport, protection of the world’s cultural and natural heritage, disaster risk reduction, urban environmental impact reduction, and building positive economic, social, and environmental links between urban, peri-urban, and rural areas. Besides Goal 11, many targets relevant to urban–rural linkages are embedded under other Goals, such as Goals 2 (food security and sustainable agriculture), 4 (equitable and quality education), 5 (gender inclusiveness), 6 (water and sanitation), 7 (energy), 8 (economic growth and decent work), 9 (sustainable infrastructure, industry, and innovation), 10 (reducing inequalities), 12 (sustainable production and lifestyles), 13 (climate actions), and 15 (terrestrial ecosystems). Serving as an integrated framework for achieving the 2030 Agenda for sustainable development and covering a broad scope of the three dimensions (economic, social, and environmental), SDGs provides an appropriate framework for analyzing the urban–rural linkages.

SDGs, through the intrinsic relationships among the goals and between the targets, forms an indivisible system. An SDG interlinkage analysis helps in understanding the relationships among the SDGs and thus informing policymaking on the potential effects of achieving one area of development on other areas. When the identified top solutions are mapped with corresponding SDG targets, an SDG interlinkage analysis can be used to analyze the interlinkages of the top solutions with other relevant SDG targets. The SDG interlinkage analysis can identify the synergies (regarding positive relationships) and trade-offs (regarding negative relationships) among the top solutions and between the top solutions and other SDG targets. This will enable customized solutions by maximizing the synergies and minimizing the trade-offs in policymaking. In this study, we applied the SDG Interlinkages Tool (V3.0) (Zhou et al. 2019) to conduct an SDG interlinkage analysis of the top solutions for building effective urban–rural linkages.

The SDG Interlinkages Tool was selected for conducting an SDG interlinkage analysis because of the following features of the tool: (1) It is comprehensive in covering all 17 goals and 169 targets at the national level for 27 countries in Asia and Africa, including Ghana. (2) It is transparent with all data available online, including the identification of the interlinkages, indicators and their time-series data, and the dashboards on the synergies and trade-offs. (3) The tool is convenient through the online visualization interface which enables to select a country, goals, and relevant targets. (4) It is flexible in modeling newly defined relationships among the 169 targets and additional elements and associated relationships beyond the 169 targets.

The SDG Interlinkages Tool was built on the following methods for the identification and quantification of the interlinkages between relevant targets at the national level (Zhou and Moinuddin 2017):

- Step I: the identification of the interlinkages between relevant SDG targets is based on causation, mainly through a comprehensive literature review together with expert opinions and a certain level of stakeholder consultations regarding specific issues.
- Step II: indicators with trackable data are selected for corresponding SDG targets based on the Global SDG Indicators (UNSD 2018) and other global indicators (e.g., the World Bank’s World Development Indicators).
- Step III: time-series data (1990–2019) are collected for the indicators in 27 countries in Asia and Africa (including Ghana) and refined for data analysis.
- Step IV: the identified SDG interlinkages (Step I) are quantified to build the SDG interlinkage model using statistical methods based on the indicator-level time-series data collected for 27 countries (Step III).

Under the SDG Interlinkages Tool, the SDG interlinkages are defined based on the causal relationships between relevant targets. A link with the direction from one target to another shows the impact from one target to the other. Quantification is based on Pearson correlation analysis, with the coefficient showing a linear relationship between a pair targets based on their historical trend. The coefficients range between [− 1, 1], with positive ones representing positive linear relations and negative ones representing negative linear relations. Coefficients with a larger absolute value (e.g., 0.9) show strong linear relationships, and those with a smaller absolute value (e.g., − 0.2) show weak linear relationships. In this study, we used positive linkages to indicate potential synergies and negative linkages to indicate potential trade-offs.

Based on the mapping of the top four solutions identified by Somanje et al. (2020) with corresponding SDG targets (Fig. 1 and Table 1), the mapped SDG targets for building effective urban–rural linkages were inputted into the Ghana’s model of the SDG Interlinkages Tool. By running the model with the inputs through the online interface, the synergies and trade-offs of the mapped SDG targets were analyzed.

### SDG indicators and data for Ghana

For the SDG Interlinkages Tool, a set of indicators of the SDG targets with trackable data was selected based on the Global SDG Indicators (UNSD 2018) and other proxy indicators, such as the World Bank’s World Development Indicators (World Bank 2019). The time-series data (1990–2019) for each indicator was collected from publicly available and internationally recognized sources. The UN Statistics Division’s SDG Indicators Global Database (UNSD 2018) is the primary data source, with other data collected from the databases provided by the World Bank (World Bank 2019) or other UN or international organizations. For Ghana, 83 indicators with trackable data were selected (Zhou et al. 2019). Table 1 shows the indicators and the corresponding data of the SDG targets that are mapped with the top four solutions in Ghana.

## Results

Using the SDG Interlinkages Tool for Ghana, the synergies and trade-offs of the top solutions for building effective urban–rural linkages were analyzed, and the results are presented as follows.

### Gender inclusiveness to strengthen urban–rural linkages: SDG synergies and trade-offs

**Fig. 2:**
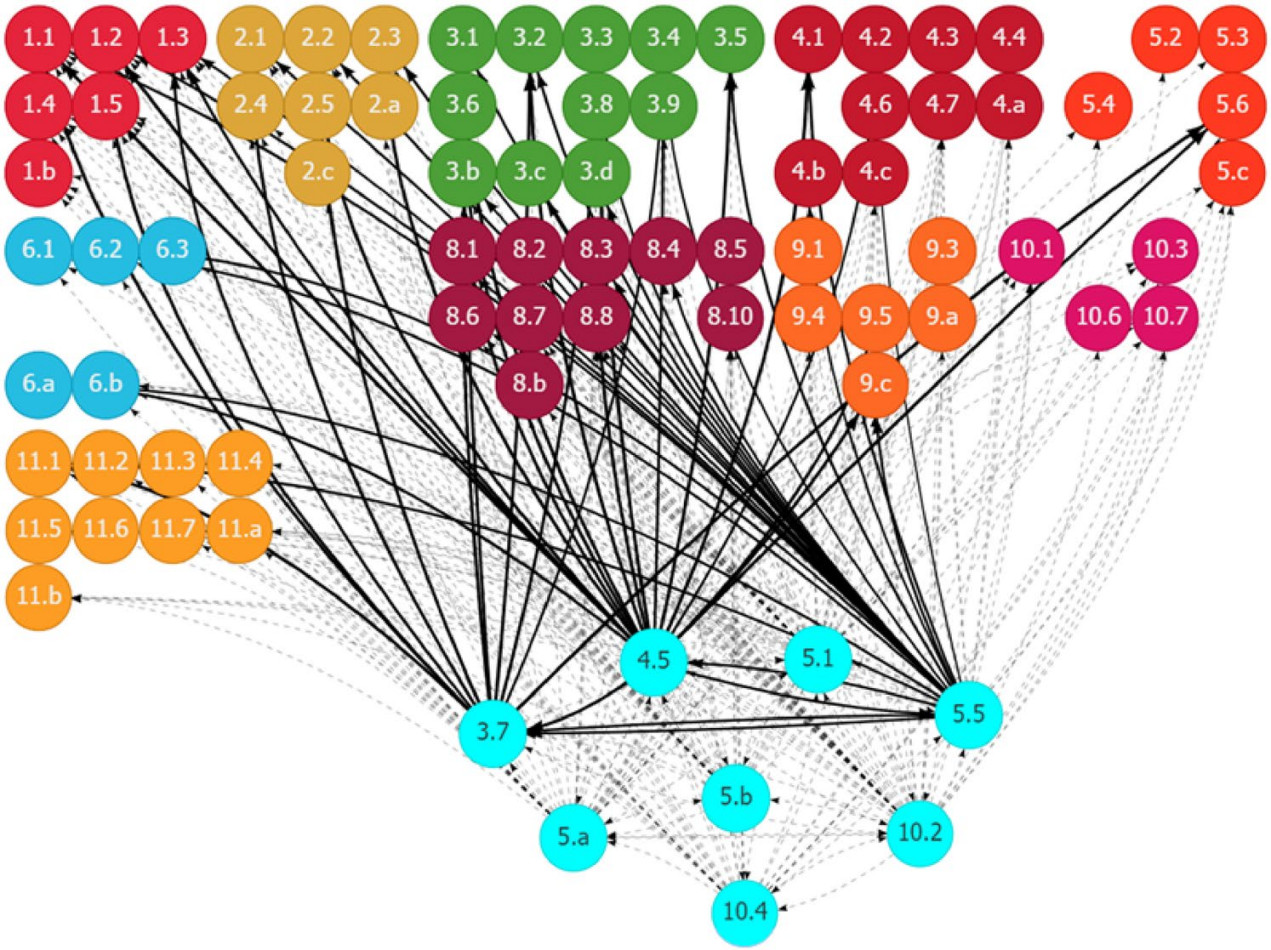
Synergies of gender inclusiveness with other SDG targets through outward linkages in Ghana. (1) Each node represents one SDG target, e.g., Target 5.1, with the fluorescent blue ones showing the selected targets. (2) Each line with an arrow linking two nodes represents a causal link between the paired targets, e.g., the line pointing from Target 5.1 to Target 4.5. (3) The value for each line (by placing the cursor over the line when using the web interface) shows the strength of the linear relationship between the paired targets. (4) The black lines represent positive linkages, and the red lines represent negative linkages. The dotted lines show that the quantification of the linkages is unavailable because of the lack of indicator-level data for the targets. (5) An outward link is the directed line pointing from the selected target to another target, e.g., the line pointing from Target 3.7 to Target 1.3 is an outward link of Target 3.7. An inward link is in the opposite direction of an outward link, directing from another target to the selected target. Source: Generated using the SDG Interlinkages Tool for Ghana (Zhou et al. 2019)

The SDG interlinkage analysis reaffirms the findings in Somanje et al. (2020) that gender inclusiveness is a crucial social element for addressing Ghana’s urban–rural dichotomy. Policies for improving gender inclusiveness generate synergistic spillover effects on several SDG areas, contributing to the sound development in both urban and rural areas. Although eight SDG targets are found to be related to gender inclusiveness (Table 1), only three of them have relevant indicators with trackable data. Figure 10 in Appendix 2 shows the historical trends of the three indicators. Figure 2 shows the synergies of the eight gender-related targets, indicated as fluorescent blue nodes at the bottom of the figure, with other SDG targets. Given Ghana’s existing SDG trends, enhancing gender inclusiveness is expected to positively affect a range of SDG goal areas and targets. These include many social targets related to poverty (Goal 1), hunger (Goal 2), health and well-being (Goal 3), quality education (Goal 4), gender and other inequalities (Goal 5 and 10), and basic services, such as access to water (Goal 6) and housing and transport (Goal 11). In the context of the existing inequalities between urban and rural areas in these social development areas, gender inclusiveness solutions could help in bridging the gaps by strengthening the positive linkages. It will also boost the economic dimension of sustainability, as evidenced by the positive links of gender-related targets with many targets related to economic growth and job creation (Goal 8), and industrial development (Goal 9).

**Fig. 3:**
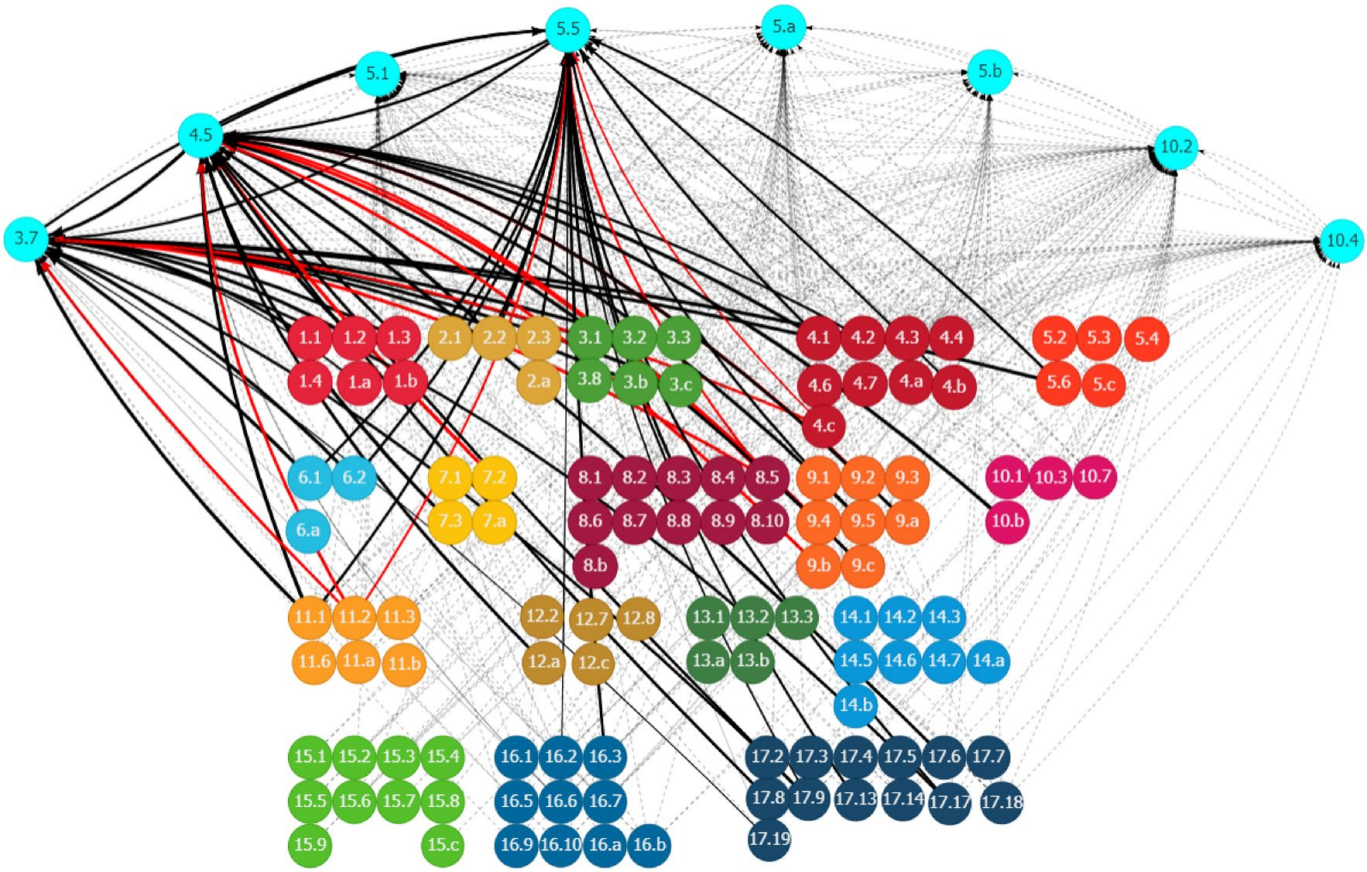
Other SDGs influencing gender inclusiveness in Ghana through inward linkages. See Fig. 2. Source: Generated using the SDG Interlinkages Tool for Ghana (Zhou et al. 2019)

Achieving gender inclusiveness also relies on other influencing factors. Figure 3 shows that progress in many other SDG goal areas could affect Ghana’s efforts to achieve gender inclusiveness, either positively or negatively. For instance, Target 5.5 on enhancing women’s participation in decision-making is reinforced by progress in ending hunger and malnutrition (Goal 2), access to reproductive healthcare services (Goal 3), reduced gender disparities in education (Goal 4), access to water, sanitation (Goal 6) and housing services (Goal 11), improved working conditions and decent work (Goal 8), improved rule of law (Goal 16), and partnerships (Goal 17), among others. Strengthening these areas would accelerate progress in achieving gender equality.

Meanwhile, certain measures could negatively affect gender-related targets (Fig. 3). For instance, given the past and current trends in Ghana, a couple of lagged areas, including industrial development, the transport sector, and employment are the development drags reducing gender disparities in education (Target 4.5) and improving women’s participation in decision-making (Target 5.5). Addressing these lagged areas could remove the drags and achieve further improvement in gender inclusiveness in Ghana.

### Investment in basic services and infrastructure to strengthen urban–rural linkages: SDG synergies and trade-offs

**Fig. 4:**
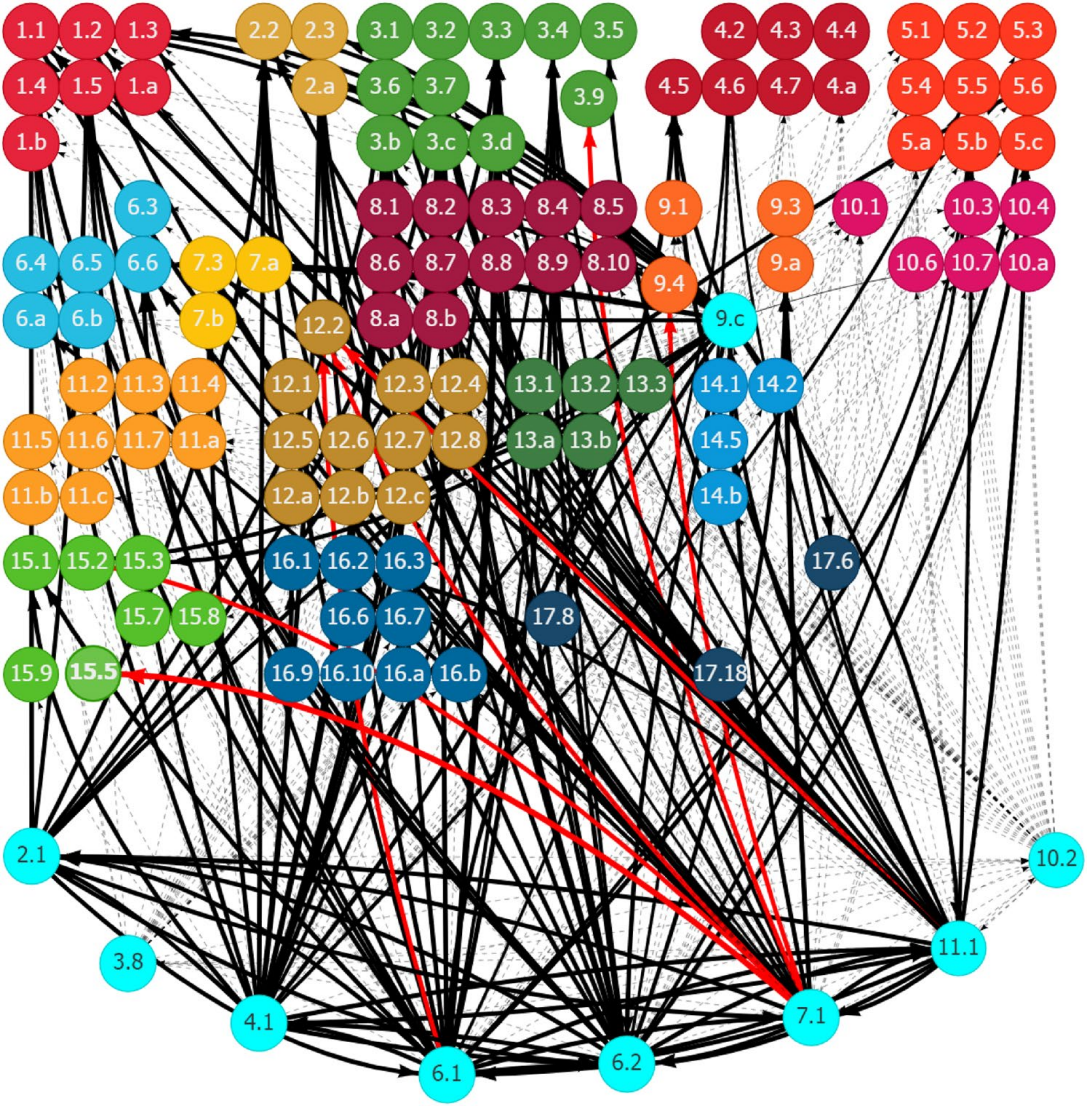
Synergies and potential trade-offs of the investment in basic and economic infrastructure with other SDG targets through outward linkages in Ghana. See Fig. 2. Source: Generated using the SDG Interlinkages Tool for Ghana (Zhou et al. 2019)

Significant urban–rural disparities are observed in basic services, such as access to food, water, energy, housing, and economic infrastructure. Nine basic-services-related SDG targets were identified for improving urban–rural linkages in Ghana (Table 1). Seven out of nine targets have relevant indicators with trackable data. Figure 11 in Appendix 2 shows the historical trend of the seven targets. Figure 4 shows that progress in the nine target areas could help in achieving several other SDG targets through positive linkages. These synergistic areas include poverty elimination (Goal 1), zero hunger (Goal 2), good health and well-being (Goal 3), quality education (Goal 4), inequalities reduction (Goal 10), economic growth and decent works (Goal 8), sustainable industrial development (Goal 9), sustainable cities (Goal 11), sustainable consumption and production (Goal 12), and good governance and partnership (Goals 16 and 17). Furthermore, it will strengthen climate actions (Goal 13). Some conflicting relationships are also observed. For instance, improved access to basic services, such as energy (Target 7.1) and housing (Target 11.1), could increase the pressure to resources use (Target 12.2) and associated emissions, endangering public health from pollution (Goal 3), and increasing industrial emissions because of fossil-based energy use (Target 9.4). Therefore, measures promoting basic services in Ghana must pay attention to these trade-offs, specifically regarding the competition for resource allocation.

**Fig. 5:**
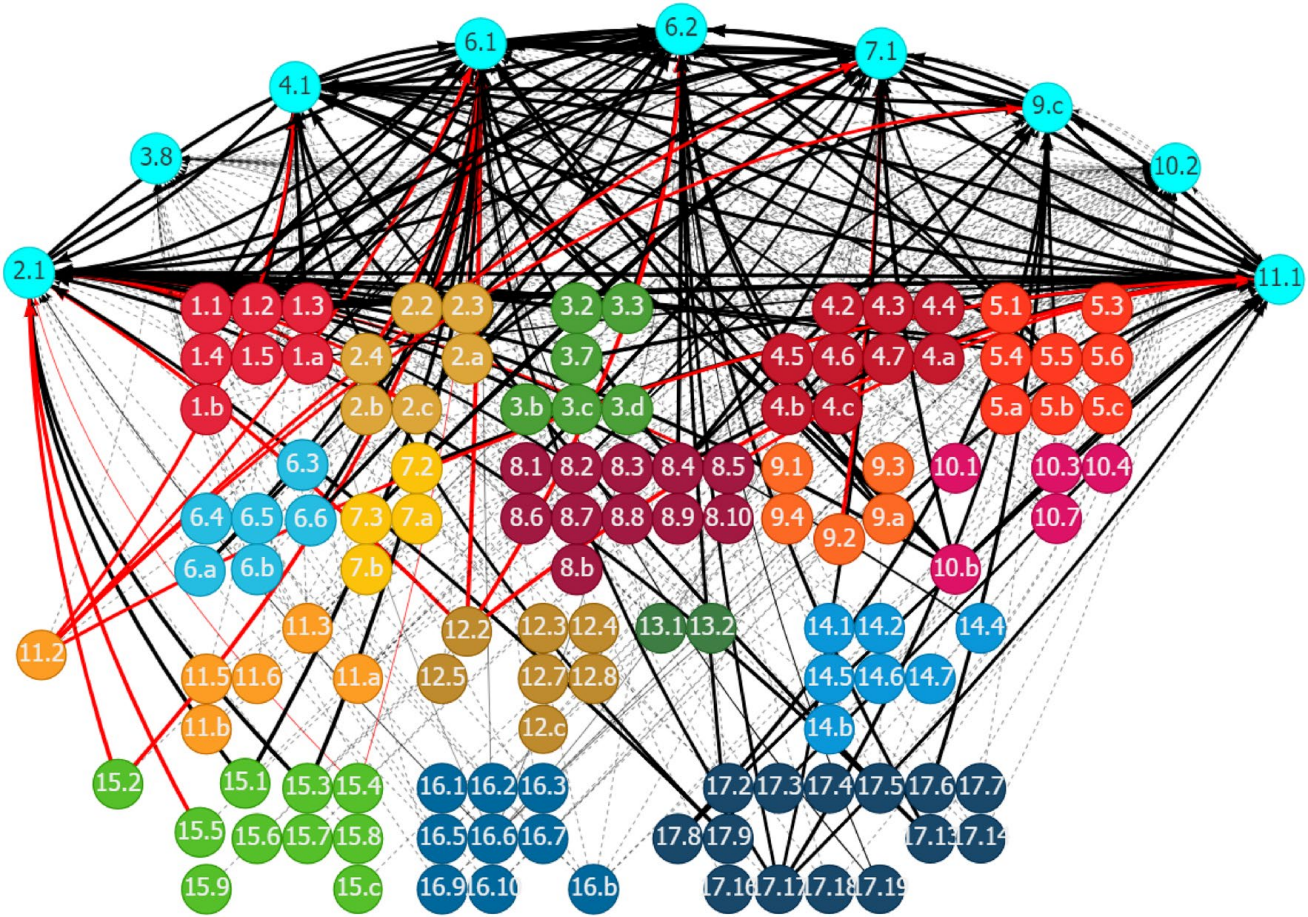
Other SDGs influencing basic services and infrastructure in Ghana through inward linkages. See Fig. 2. Source: Generated using the SDG Interlinkages Tool (Zhou et al. 2019)

Basic services also depend on the progression of other SDGs, which is captured in the inward linkages (Fig. 5). For example, Targets 6.1 and 6.2 (access to water and sanitation) are positively affected by measures to reduce poverty (Goal 1) and hunger (Goal 2), improved disease control (Goal 3), enhanced access to education (Goal 4), women’s participation in education (Goal 5), increased access to energy (Goal 7) and housing (Goal 11), inclusive growth, economic productivity and efficiency (Goal 8), access to information and communication technology (Goal 9), official development assistance (ODA) (Goal 10), sustainable use of freshwater, improved rule of law (Goal 16), and measures for promoting sustainable development (Goal 17). Strengthening these positive linkages will accelerate the progress in universal access to basic services and infrastructure.

However, several SDG targets could negatively affect the promotion of basic services and infrastructure through negative inward linkages. For instance, inadequate and inefficient transport systems (11.2), degradation in relevant ecological systems, and unsustainable resource use (12.2) in Ghana are causing a scarcity of resources and fiercer competition among the sectors dependent on these resources. This, in turn, generates a dragging effect on the expansion of basic services and related infrastructure. Addressing these lagged areas could remove the drags and release the potential for making further progress in the provision of basic services and infrastructure in Ghana.

### Development of sustainable agriculture systems to strengthen urban–rural linkages: SDG synergies and trade-offs

Agriculture is a critical export sector in Ghana, with cocoa accounting for 25% of its total exports and approximately 20% of global cocoa exports (World Bank 2018). Despite the importance of agriculture as an engine to achieve overall economic growth and recovery, the value-added share of agriculture in GDP has fallen substantially from almost 45% in 1990 to approximately 18% in 2018 (World Bank 2019). These figures reflect the challenges in addressing low productivity dominated by primary production with limited agro-processing and value addition, low governmental support indicated by low governmental expenditure in the agriculture sector (approximately 4% in 2017), and low foreign direct investment of less than 0.1% in 2017 (Government of Ghana 2019; World Bank 2018). These have directly and indirectly affected food security, improved nutrition, urban–rural linkages, and other aspects of sustainable development in Ghana.

**Fig. 6:**
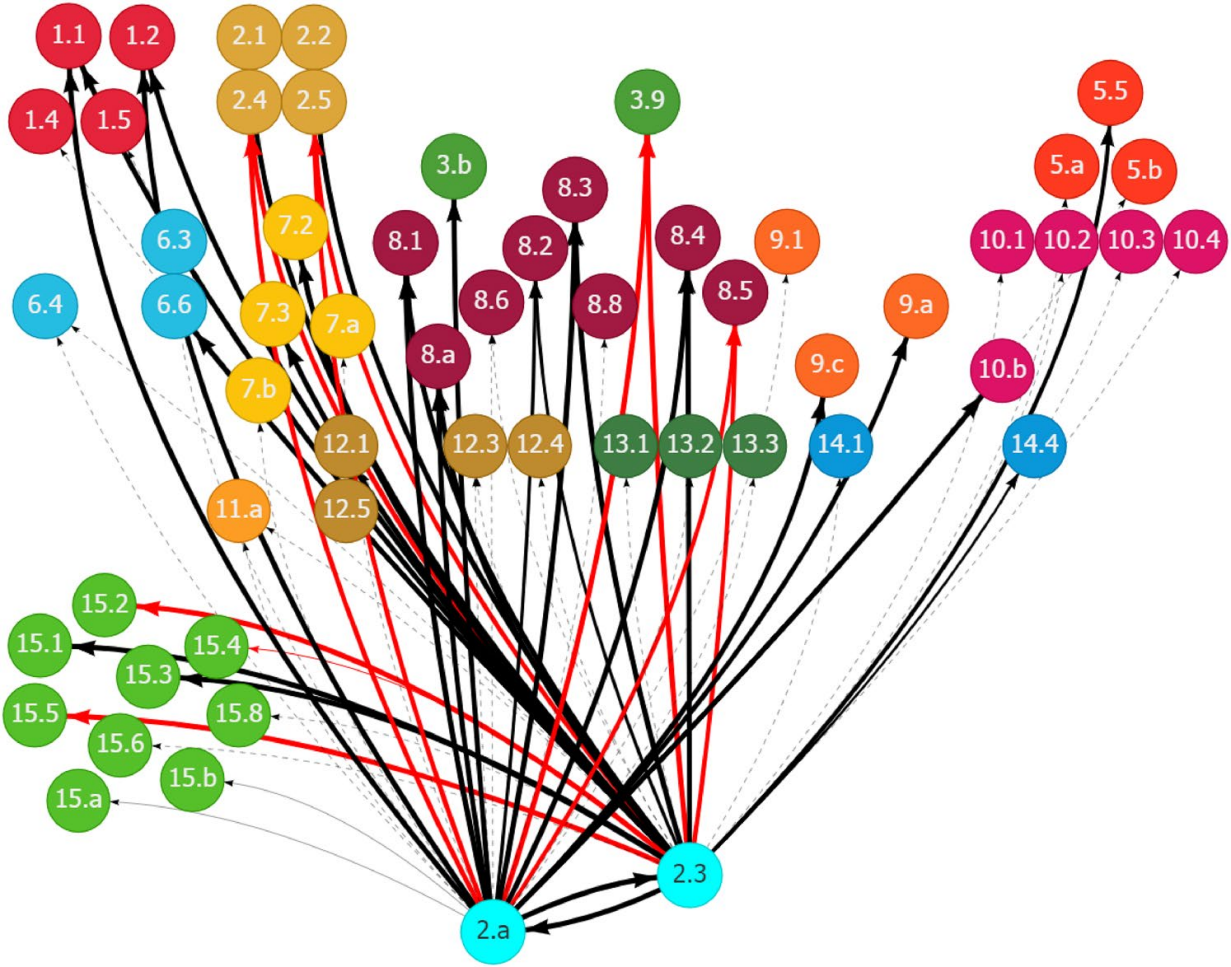
Synergies and potential trade-offs of the development of sustainable agriculture systems with other SDGs through outward linkages in Ghana. See Fig. 2. Source: Generated using the SDG Interlinkages Tool for Ghana (Zhou et al. 2019)

SDG Targets 2.3 and 2.a were mapped with the top solution on the promotion of sustainable agriculture systems in Ghana (Table 1). On the basis of the SDG interlinkage analysis, improving agriculture productivity (Target 2.3) and increasing the investment in rural infrastructure and agricultural extension services (Target 2.a) could generate synergies with some social and economic development areas but at the same time have potential trade-offs, specifically with the environment. Figure 12 in Appendix 2 shows the historical trend of the corresponding SDG targets to promote sustainable agriculture systems. On the basis of the historical trend in related interactions, Fig. 6 shows that boosting agriculture productivity and investing in rural infrastructure and extension services contributed positively to eradicating poverty and building the resilience of the poor (Goal 1), reducing hunger and improving nutrition (Goal 2), enhancing economic growth and productivity and creating decent jobs (Goal 8), and the development of resilient infrastructure (Goal 9). However, improving agriculture productivity and increasing the investment in rural infrastructure and extension services would increase chemical inputs (Target 2.4), damage genetic diversity (Target 2.5), and cause problems in public health (Target 3.9), loss of labor productivity (Target 8.5), and ecological degradation, including forests (Target 15.2), mountain ecosystems (Target 15.4), and biodiversity (Target 15.5). Promoting agriculture development in Ghana through productivity improvement and building sustainable and resilience production systems is critical to enhance the ecological resilience of urban–rural linkages.

**Fig. 7:**
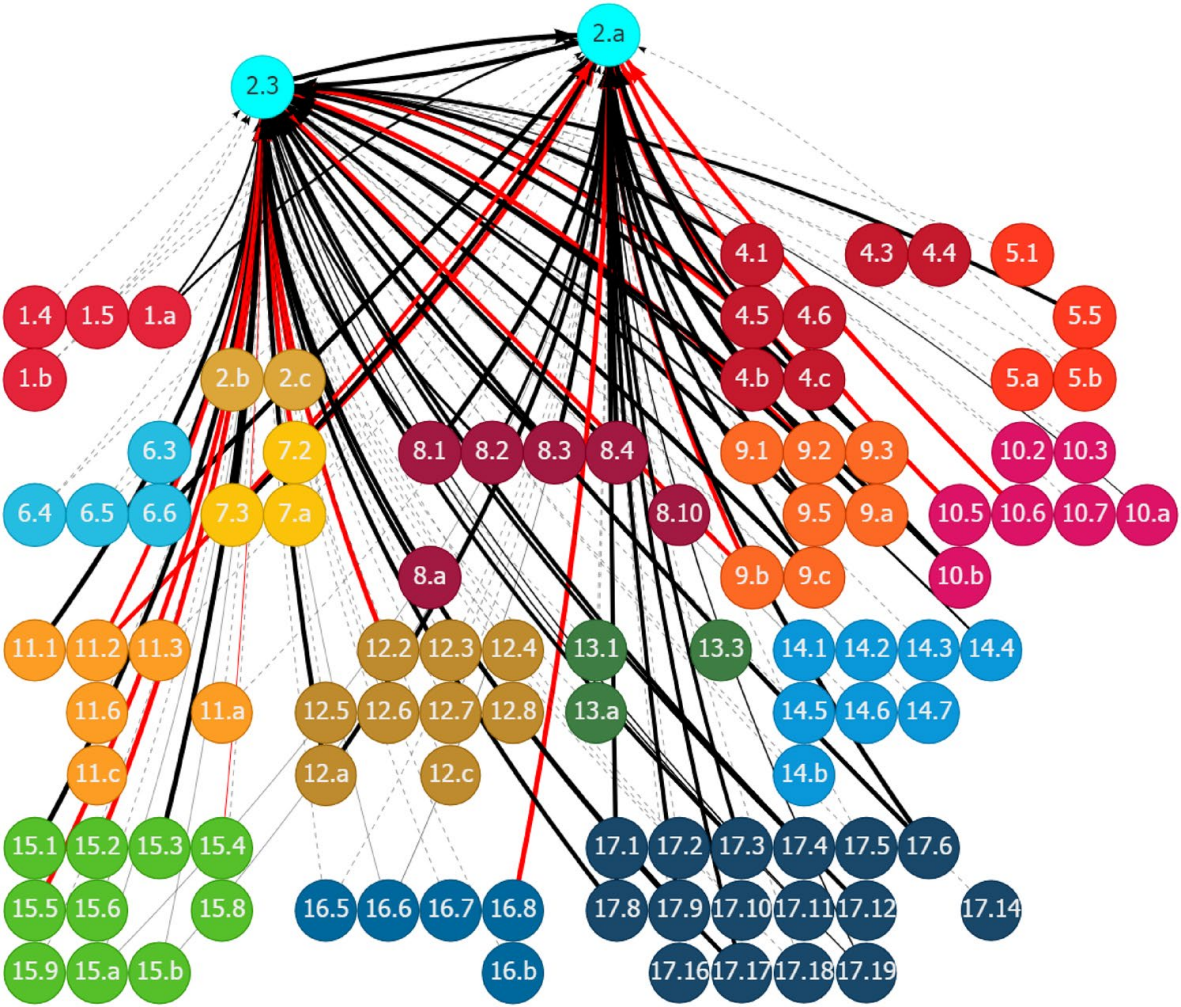
Other SDG targets influencing the development of sustainable agriculture systems in Ghana through inward links. See Fig. 2. Source: Generated using the SDG Interlinkages Tool for Ghana (Zhou et al. 2019)

Several factors related to the development in other SDG areas could affect the effectiveness of promoting sustainable agriculture systems in Ghana, either positively or negatively. The SDG interlinkage analysis presents these factors through inward linkages (Fig. 7). Factors contributing positively to the promotion of sustainable agriculture systems in Ghana include improving equality in education, specifically equal access to all levels of education to all (Target 4.5), reducing gender inequalities (Target 5.5), increasing the aid for trade and ODA for least developed countries (Targets 8.a and 10.b), enhancing economic productivity and resource use efficiency (Targets 8.2 and 8.4), promotion of resilient infrastructure and enhancement in R&D (Targets 9.a and 9.5), improving access to financial services to small and medium-sized enterprises (Target 9.3), and building accountable governance and multi-stakeholder partnerships (Goals 16 and 17). Strengthening these areas will accelerate progress in promoting sustainable agriculture systems. Areas that could negatively affect building sustainable agriculture systems in Ghana include the lack of properly functioning food commodity markets (Target 2.c), insufficient transportation infrastructure (Target 11.2), unsound industrial development regarding the lack of industrial diversification and value addition (Targets 9.2 and 9.b), weak voice from developing countries in decision-making in international economic and financial institutions (Targets 10.6 and 16.8), and ecological degradation in the areas of forests and biodiversity (Targets 15.2, 15.4, and 15.5). Addressing these lagged areas could remove the development drags and release the potential for further development in building sustainable agriculture systems in Ghana.

### Promotion of innovative financial systems to boost urban–rural linkages: SDG synergies and trade-offs

The promotion of innovative and inclusive financial systems to enable universal access by providing financial services, such as credits, crop insurance, and channels of money transfer for smooth remittance and cash flow, could enhance people’s involvement in economic and social activities, thus ensuring financial security and economic and political stability (Somanje et al. 2020).

**Fig. 8:**
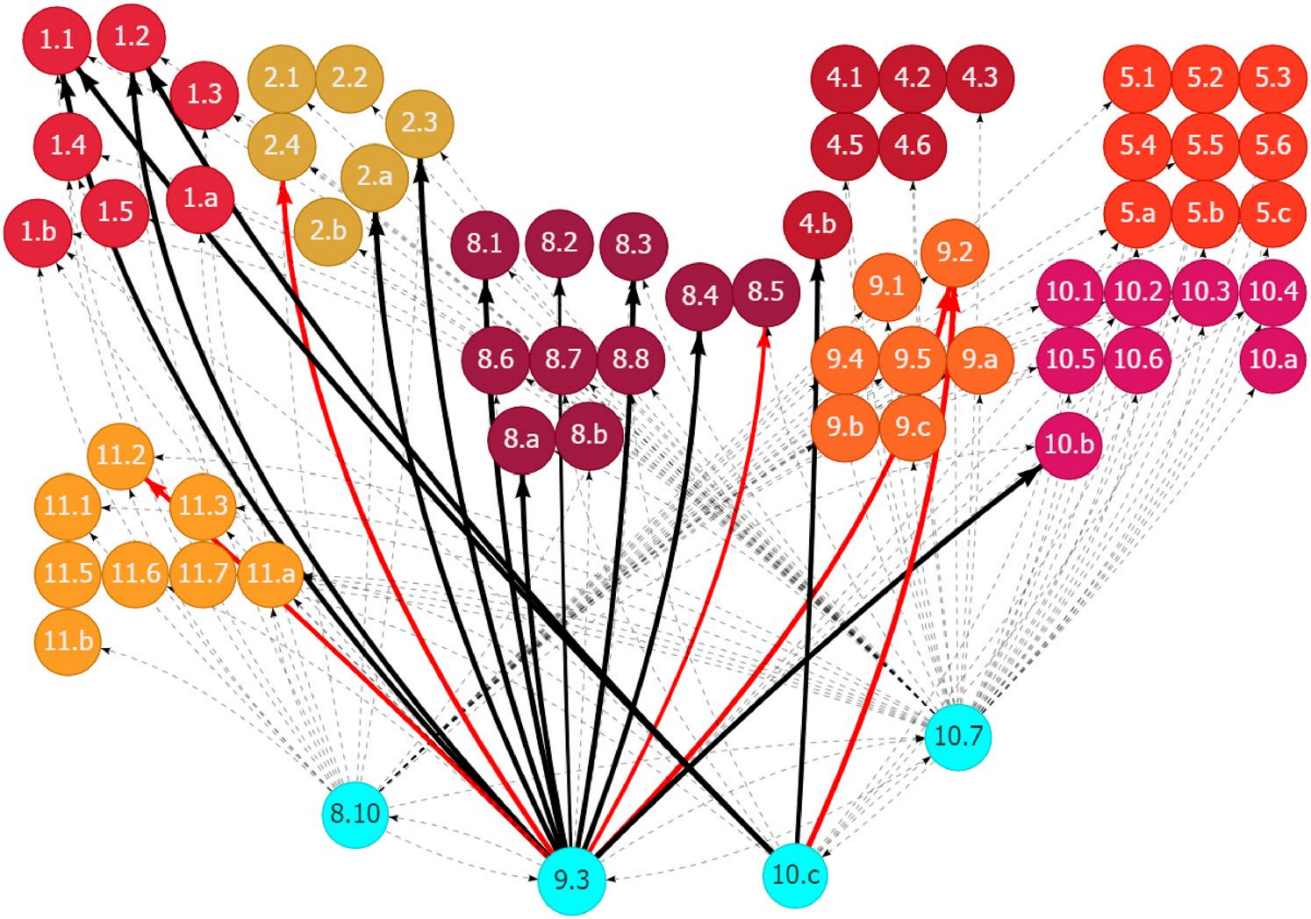
Synergies and potential trade-offs of promoting innovative financial inclusion systems with other SDG targets through outward linkages in Ghana. See Fig. 2. Source: Generated using the SDG Interlinkages Tool for Ghana (Zhou et al. 2019)

For the interlinkage analysis, four SDG targets are identified corresponding to the promotion of innovative financial inclusion systems in Ghana (Table 1). Three of the four targets have relevant indicators with trackable data (Fig. 13 in Appendix 2). The SDG interlinkage analysis (Fig. 8) shows the synergistic effects of promoting innovative financial inclusion systems by increasing the access of small-scale enterprises to financial services (Target 9.3) and reducing the transaction costs of migrant remittances (Target 10.c) on many social and economic sectors in Ghana. These sectors include poverty eradication (Targets 1.1 and 1.2); boosting agriculture productivity and the investment in rural infrastructure and extension services (Targets 2.3 and 2.a); enhancing economic growth, productivity, and resource efficiency improvement (Targets 8.1, 8.2 and 8.4); formalization and growth of small and medium-sized enterprises (Target 8.3); increasing aid for trade (Target 8.a); and ODA (Target 10.b) to least developed countries. However, promoting access to financial systems, specifically to the agriculture and industrial sectors, must consider the potential negative impacts on the labor market (Target 8.5), industrial value addition (Target 9.2), and the environment through chemical inputs of agriculture development (Target 2.4). Furthermore, financial inclusion systems should be directed to the promotion of investment in the development of relevant transport systems and infrastructure (Target 11.2), which presents as a constraint to the further development in the agriculture and industrial sectors in Ghana.

**Fig. 9:**
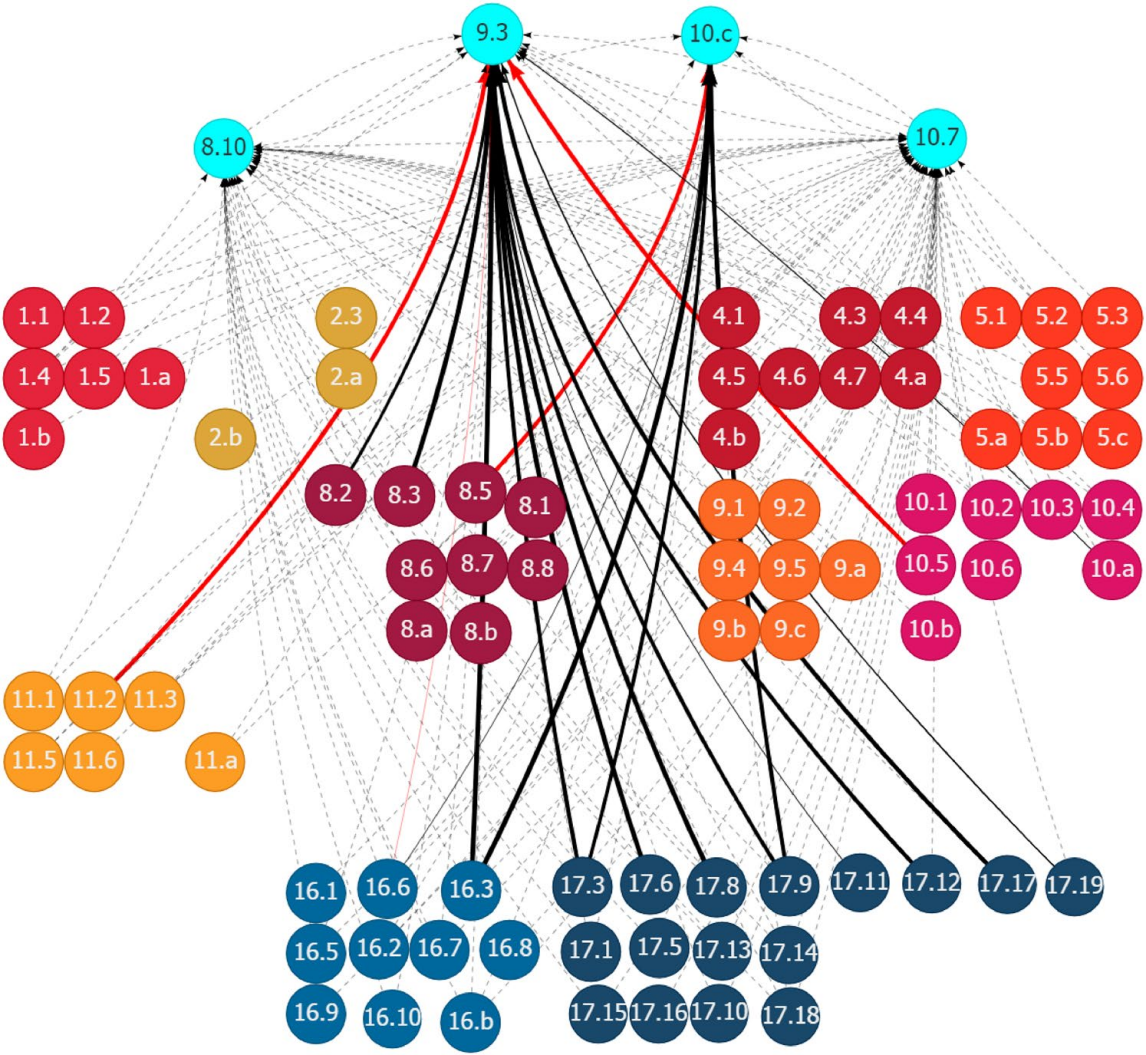
Other SDG targets influencing the promotion of innovative financial inclusion systems in Ghana through inward linkages in Ghana. See Fig. 2. Source: Generated using the SDG Interlinkages Tool for Ghana (Zhou et al. 2019)

**Fig. 10:**
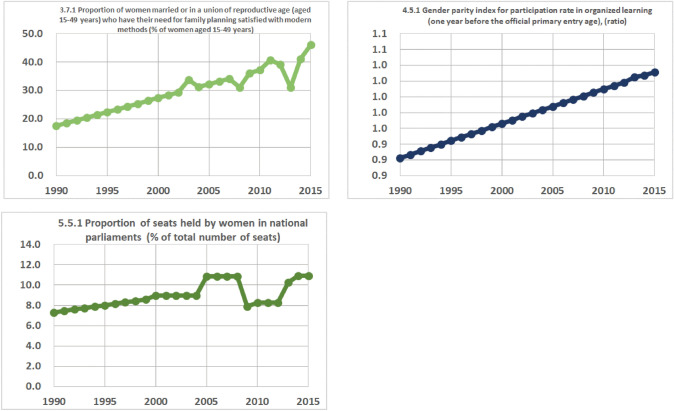
Historical trend of the corresponding SDG targets related to gender inclusiveness in Ghana. Source: The SDG Interlinkages Tool (Zhou et al. 2019)

**Fig. 11:**
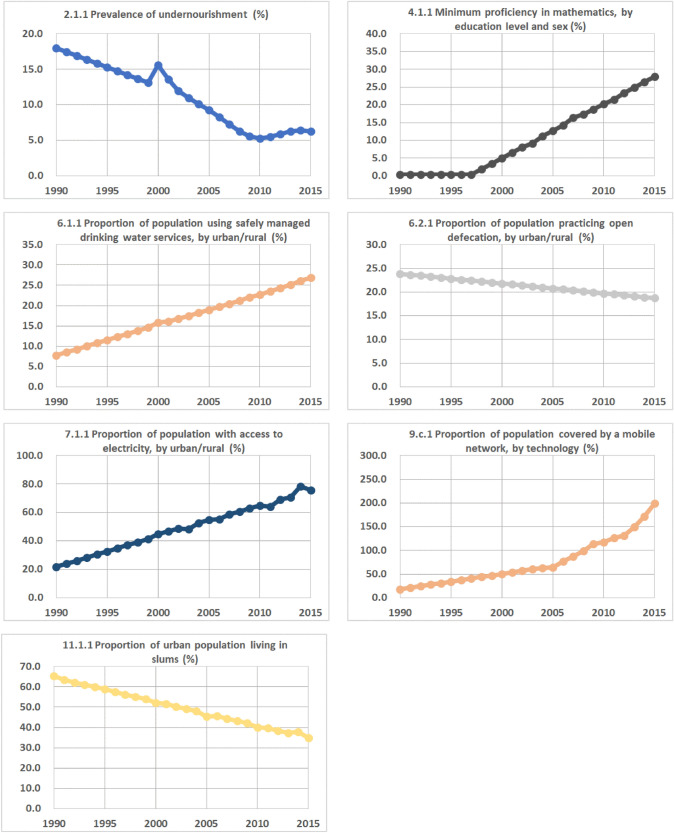
Historical trend of the corresponding SDG targets related to investment in basic and economic infrastructure in Ghana. Source: The SDG Interlinkages Tool (Zhou et al. 2019)

**Fig. 12:**
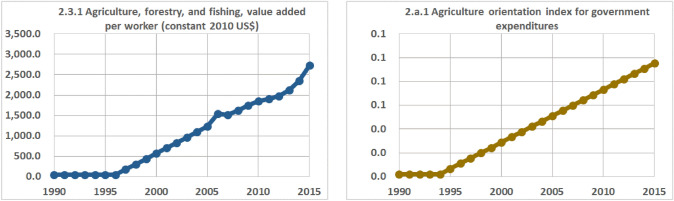
Historical trend of the corresponding SDG targets related to the development of sustainable agriculture systems in Ghana. Source: The SDG Interlinkages Tool (Zhou et al. 2019)

**Fig. 13:**
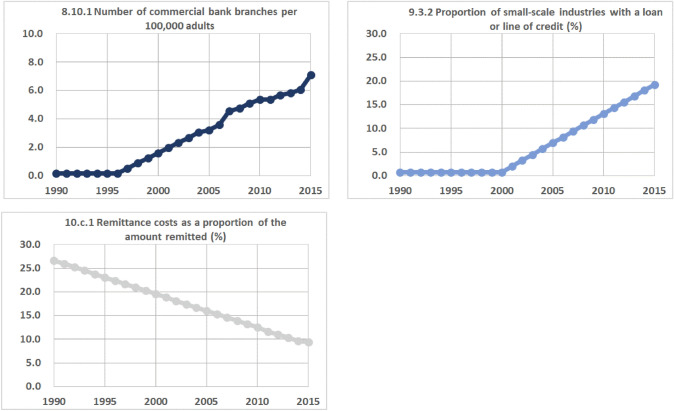
Historical trend of the corresponding SDG targets related to the promotion of innovative financial inclusion systems in Ghana. Source: The SDG Interlinkages Tool (Zhou et al. 2019)

Certain factors related to the development in other SDG areas could affect the effectiveness of the promotion of innovative financial inclusion systems in Ghana, either positively or negatively (Fig. 9). The influencing factors, through positive inward linkages toward Targets 9.3 and 10.c, include those for enhancing economic productivity and the formalization and growth of small- and medium-sized enterprises (Targets 8.2 and 8.3), strengthening governance (Target 16.3 on the rule of law) and partnership, including mobilizing financial resources and providing other supports to developing countries (Targets 17.3 and 17.9), enhancing international cooperation on access to science and technology (Target 17.6), increasing the experts of developing countries (Targets 17.11 and 17.12), and promotion of public, private, and civil–social partnership (Target 17.17). Strengthening the progress in these areas accelerates progress in promoting innovative financial inclusion systems in Ghana. However, a few areas related to the unemployment rate (Target 8.5), insufficient development of transport infrastructure (Target 11.2), and lack of effective regulation and monitoring of financial markets (Target 10.5) could drag the development in financial inclusion systems. Addressing these lagged areas could release the potential for building innovative financial inclusion systems in Ghana.

## Discussion

### Top solutions and implications for sustainable urban–rural development in Ghana

The analysis shows that progress in gender inclusiveness and improved access to basic services could significantly address the urban–rural dichotomy in Ghana. Improved gender inclusiveness will positively influence in transforming society by contributing to reducing poverty and hunger, improving health and education, addressing inequalities, and enhancing access to basic services. Developing tailored programs that address the needs of women and girls is critical for their empowerment (Somanje et al. 2020). Evidence has shown a strong correlation between female education and poverty reduction in developing countries (United Nations Women 2014). Given that gender inequality is endemic, particularly in northern Ghana, mainstreaming gender inclusiveness in decision-making processes would be critical in bridging the gap. The current free senior high school initiative needs sustainable funding resource to make it permanent. The initiative needs to be tailored closely with girl-child education, as it has every potential to enhance gender inclusiveness by improved access to education, which is critical for reducing the existing educational inequalities between rural and urban areas. However, gender inclusiveness will not be achieved automatically because it also depend on progress in other areas. Progress in water, sanitation, and housing services, and an improved rule of law, for example, could enhance women’s participation in decision-making. This can improve decentralized service provisions and strengthen regional and local governmental offices to be more effective in the discharge of their duties. This is important given that weak urban–rural linkages in Ghana is partly attributed to the ineffectiveness of decentralization at the regional and district levels (Owusu 2005). The National Commission for Civic Education (NCCE) and the gender ministry need to be empowered, particularly at the district level, to develop gender-sensitive educational and training programs for women and girls on various issues, such as livelihood, civic rights, and nutrition.

The SDG synergy and trade-off analysis on the improvement of agriculture productivity and increase in investment in extension services shows that enhancing agriculture productivity will help in reducing poverty in rural areas and contribute to ending hunger, improving nutrition in both rural and urban areas, and boosting nation-wide economic development. Although this is consistent with the wider agriculture productivity and poverty reduction literature (e.g. Benin et al. 2008; Ravallion 2009), Ghana specific study shows that a significant increase in productivity is needed to accelerate poverty reduction (Dzanku 2015). Given that the agricultural sector in Ghana is dominated by small scale holders, improving productivity would require tailored interventions, such as providing incentives to farmers (e.g. soft loans, free seeds, extension training programs), strengthening the value-chain and scholarship packages to all farmers (not only cocoa farmers). Meanwhile, without proper natural resources management and pollution control policies in place, rural development by enhancing agriculture productivity could negatively affect genetic diversity and biodiversity, and public health by increased chemical use and release into the environment. Integrated planning is indispensable for achieving sustainable urban–rural development. Investment in agriculture productivity enhancement, processing and extension services, and relevant infrastructure such as transport systems to enhance the urban–rural linkages will have lasting consequences on the livelihoods of relevant communities in both rural and urban areas. Importantly, the government has to commit to the current developmental projects, especially the one-district-one-factory (1D1F), one-village-one-dam, the feeder roads and planting for food, as these have the potential to not only improve agricultural productivity but also foster effective urban–rural linkages.

The synergy and trade-off analysis on the promotion of innovative financial inclusion systems suggests that reducing inequalities of access to financial services in rural and urban areas, formal and informal sectors, and enterprises of different sizes could generate multiple spillover effects. It will impact positively on poverty eradication, agriculture productivity improvement, economic growth enhancement, productivity and resource efficiency improvement, formalization and growth of small- and medium-sized enterprises, and increased aid for trade and ODA to least developed countries. These impacts will benefit both rural and urban areas. In the case of Ghana, promoting and strengthening mobile money usage and rural banking would be critical in enhancing financial inclusion and contribute to achieving sustainable development (National Development Planning Commission [NDPC] 2017). Given that mobile money (popularly called ‘Momo’) platforms may be used for fraudulent activities, strict regulation and monitoring would be important to sanitize the sector and ensure smooth operation.

### Potentials and future research agenda of an SDG interlinkage analysis

This study integrates the solution-scanning approach and SDG interlinkage analysis to investigate effective solutions for building sustainable urban–rural linkages in Ghana. This study extends the area of solution scanning with a further assessment of the screened solutions regarding the synergies and trade-offs from an SDG interlinkage perspective. The added value of this study to the area of SDG interlinkage analysis is to enable a problem-and-solution-driven exercise through which stakeholder consultations provide invaluable inputs to screen the top solutions and map with SDG targets. Identify key points (leverage points) to maximize synergies and minimize potential trade-offs were identified for policy formulation.

An SDG interlinkage analysis, either qualitatively or quantitatively, could provide a comprehensive overview of the interactions among the key elements of the human–environment system and, therefore, it supports a systems approach to problem-solving. It is a powerful tool to help in addressing development issues, such as sound urban–rural development, which cut across multiple spheres of the social, economic, and environmental dimensions. An SDG interlinkage analysis can identify and analyze the potential synergies and trade-offs that help in informing effective priority setting and resource allocation. For example, in building effective urban–rural linkages in Ghana, enhancing gender inclusiveness and improving basic services and infrastructure could be considered together rather than siloed issues because of the many synergies among the improvement in the two areas. By addressing the two issues together, limited financial and human resources can be used more effectively than addressing the two issues separately.

Furthermore, understanding the trade-offs of the proposed solutions is critical to avoid lock-in effects in unsustainable pathways and ensure policy integrity and coherence. For example, promoting agriculture productivity improvement to strengthen the urban–rural linkages in Ghana should consider its trade-offs on environmental pollution and associated damages to public health, in both rural and urban areas, and biodiversity loss. Proper pollution prevention and control measures and sustainable agriculture practices are needed through collaboration between urban and rural areas.

For future research in the area of SDG interlinkage analysis, multi-stakeholder consultations and engagement should be strengthened in the processes of problem identification, solution screening, identification of the SDG interlinkages, enhancement of indicators, data availability, and calibration of the results and acting on the results. In this study, multi-stakeholder contributions are limited to the screening and ranking of top solutions and SDG mapping in the context of Ghana.

The availability of effective indicators and trackable data presents a major challenge to current and future research of an SDG interlinkage analysis, specifically quantitative analysis. For example, under the existing SDG Interlinkages Tool, which was used for the interlinkage analysis of the urban–rural relationships in this paper, among the 169 targets, only 83 have corresponding indicators with trackable data for Ghana. Showed as dotted lines in the visualization results, many linkages cannot be quantified. The big gaps in effective indicators and data availability have constrained conducting a full-fledged quantitative assessment. Improved and localized indicators and data availability can improve the robustness of the quantitative analysis.

## Conclusion

This study used an integrated approach by combining the results from a solution-scanning exercise with an SDG interlinkages analysis to assess the synergies and trade-offs of the identified top solutions for achieving effective urban–rural linkages in Ghana. Ghana needs to promote effective urban–rural linkages if the country is to achieve the SDGs. Ignoring such linkages will severely inhibit growth and widen spatial inequalities (World Bank 2006), which will plunge many people, particularly the vulnerable groups, into extreme poverty. Urban–rural linkages play an important role in employment creation, income generation and wealth accumulation. From an SDG interlinkages perspective, we concluded that an integrated approach is crucial for effective policymaking that enables to take advantage of the synergies and address the trade-offs of the proposed policy solutions. Putting rural and urban areas into strategic planning units will be critical in providing the enabling environment for effective trade networks and knowledge exchange between the two boundaries (Akkoyunlu 2015). Although leveraging the synergies accelerates the progress in SDGs, it is equally critical to address the trade-offs to release the chocking points and developmental drags, and thus ensure the much-needed policy integrity that is indispensable for addressing Ghana’s urban–rural divide. Lastly, like in all other countries, Ghana will also face critical challenges from the fallout of emerging issues, especially the 2020 novel coronavirus pandemic, which has already affected every aspect of human life in every country around the world. Although well managed in Ghana so far, a crisis of this scale could disproportionately affect the vulnerables and could widen the urban–rural disparity. To avoid locking in a deep disparity for a prolonged period, assessing Ghana’s COVID-19 recovery efforts and plans, and their implications for urban–rural linkages provides a critical research agenda that scholars and policymakers must prioritize.

## Appendix 1 List of 17 sustainable development goals (SDGs)

**Table:**

GOAL 1: No poverty
GOAL 2: Zero hunger
GOAL 3: Good health and well-being
GOAL 4: Quality education
GOAL 5: Gender equality
GOAL 6: Clean water and sanitation
GOAL 7: Affordable and clean energy
GOAL 8: Decent work and economic growth
GOAL 9: Industry, innovation and infrastructure
GOAL 10: Reduced inequality
GOAL 11: Sustainable cities and communities
GOAL 12: Responsible consumption and production
GOAL 13: Climate action
GOAL 14: Life below water
GOAL 15: Life on land
GOAL 16: Peace and justice strong institutions
GOAL 17: Partnerships to achieve the goal

## Appendix 2

See Figs. 10, 11, 12 and 13.
